# 86808 Adverse Childhood Experiences are associated with a higher prevalence of asthma among adolescents with sickle cell disease

**DOI:** 10.1017/cts.2021.489

**Published:** 2021-03-30

**Authors:** Brandi Pernell, Vishnu Nagalapuram, Chee Paul Lin

**Affiliations:** 1 University of Alabama at Birmingham, School of Medicine – Pediatrics; 2 University of Alabama at Birmingham, School of Medicine; 3 University of Washington

## Abstract

ABSTRACT IMPACT: This research highlights Adverse Childhood Experiences as a potential risk factor and intervention target contributing to the disproportionate number of individuals with sickle cell disease affected by asthma, a well-established catalyst to the increased morbidity and mortality impacting this high-risk population. OBJECTIVES/GOALS: Adverse Childhood Experiences (ACEs) are strongly associated with asthma. A disproportionate number of individuals with sickle cell disease (SCD) also have asthma. Asthma is strongly associated with increased SCD morbidity and mortality. This study compared the prevalence of asthma among children and adolescents with SCD with and without ACEs. METHODS/STUDY POPULATION: This retrospective cohort study involved 45 children and 30 adolescents with SCD. ACEs were captured using the Center for Youth Wellness Adverse Childhood Experiences Child and Teen Questionnaires, which encompass the original 10 ACEs as well as 7 (child) and 9 (teen) expert-recommended ('expanded') ACEs. ACE exposures were categorized as: Original 0-1 vs. ≥2; Original + Expanded 0-1 vs. ≥2. Asthma prevalence was compared among ≥2 and 0-1 ACE groups using the chi-square (or Fisher’s exact) test. A binary logistic regression was performed to predict the likelihood of asthma while adjusting for characteristics (age, household income and gender) that were statistically different among ACE comparison groups at baseline. RESULTS/ANTICIPATED RESULTS: Among the 45 child participants, 64% had a history of asthma; whereas 50% of teens had a history of asthma. Asthma prevalence was higher among teens with ≥2 vs. 0-1 Original ACEs (89% v. 33%, p=0.014). A history of ≥2 ACEs remained significant (p=0.024) among teens after adjusting for age, household income and gender. There was no significance in asthma prevalence among child ACE comparison groups. DISCUSSION/SIGNIFICANCE OF FINDINGS: Adolescents with ≥2 ACEs had a higher prevalence of asthma compared to subjects with 0-1 ACE. This study, coupled with the cumulative nature of ACEs and the graded-dose response relationship between ACEs and poor health outcomes, highlight the need for larger, longitudinal studies examining the relationship between ACEs, asthma and SCD outcomes.

